# EA-CNN: Enhanced attention-CNN with explainable AI for fruit and vegetable classification

**DOI:** 10.1016/j.heliyon.2024.e40820

**Published:** 2024-11-30

**Authors:** Zeshan Aslam Khan, Muhammad Waqar, Khalid Mehmood Cheema, Ali Abu Bakar Mahmood, Quratul Ain, Naveed Ishtiaq Chaudhary, Abdullah Alshehri, Sultan S. Alshamrani, Muhammad Asif Zahoor Raja

**Affiliations:** a International Graduate Institute of Artificial Intelligence, National Yunlin University of Science and Technology, 123 University Road, Section 3, Douliou, Yunlin, 64002, Taiwan, ROC; b Department of Electronic Engineering, Fatima Jinnah Women University, Rawalpindi, Pakistan; c Department of Electrical and Computer Engineering, International Islamic University, Islamabad, 44000, Pakistan; d Department of Business Management, Global Banking School, London, United Kingdom; e Future Technology Research Center, National Yunlin University of Science and Technology, 123 University Road, Section 3, Douliu, Yunlin, 64002, Taiwan, ROC; f Department of Information Technology, Faculty of Computing and Information, Al-Baha University, Alaqiq, 65779-7738, Saudi Arabia; g Department of Information Technology, College of Computer and Information Technology, Taif University, Taif, 21944, Saudi Arabia

**Keywords:** Explainable AI, Deep learning, Classification, Convolutional neural networks

## Abstract

The quality of vegetables and fruits are judged by their visual features. Misclassification of fruits and vegetables lead to a financial loss. To prevent the loss, superstores need to classify fruits and vegetables in terms of size, color and shape. To improve the accuracy of models for classifying fruits and vegetables, researchers have introduced various CNN architectures like VGG16, AlexNet, DenseNet-121 and different attention-based variants by compromising models' complexity leading to high computational cost and overlooking feature's contribution for interpretable predictions. Additionally, the major drawback in most of the existing works is the utilization of the limited dataset and number of classes for fruit and vegetable classification task instead of the benchmark dataset like Fruit-360 which comprises of 141 distinct fruit and vegetable classes. In this study, an explainable artificial intelligence (XAI) driven enhanced attention-CNN (EA-CNN) is proposed for accomplishing the fruit and vegetable classification task accurately and efficiently. The proposed EA-CNN model (a) detects the class of fruits and vegetable accurately through visual features by manipulating undiscover customized pooling technique and enhanced attention feature extraction mechanism, (b) classifies the fruits and vegetables efficiently through a simplified architecture, (c) provides interpretable predictions through XAI approach. By utilizing entire fruit-360 database, this research noteworthily enhances the model's capability to classify wide range of fruits and vegetables, thus providing an effective and reliable solution for practical applications. The proposed study outclasses baseline models with regard to accuracy and computational cost on fruit-360 benchmark dataset. EA-CNN achieves better accuracy of 98.1 % in smaller number of iterations as compared to benchmark models. Moreover, to further prove the efficiency, generalization ability, robustness, scalability and adaptability of this research, the architecture of proposed EA-CNN model is validated on another real-world dataset named as ‘Fruit Recognition’. The experimental outcomes display that the proposed EA-CNN model produces substantial performance on Fruit-Recognition dataset as well by attaining a generalized accuracy of 96 %.

## 1 Introduction

**Fig. 1:**
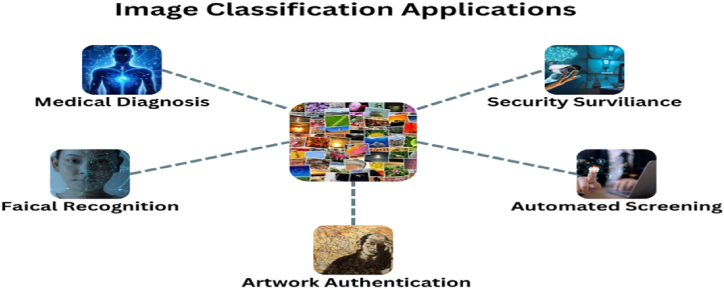
Applications of image classification.

Deep learning emerged as a significant tool of artificial intelligence [1], enabling machines to learn fast and make better decisions from complex/difficult data. The deep learning methods/algorithms allow us to effectively extract the important and useful features from the input image, thus motivating the researchers to implement the deep learning models for better solution of classification problems. The deep learning models or algorithms established their worth in solving variety of challenging classification problems arising in spectrum of applications, such as, recommendation systems [2], medical diagnosis [3], artwork authentication [4], facial recognition [5], sentiment analysis [6], and security surveillance [7]. The graphical description highlighting the importance of deep learning models in different applications is presented in Fig. 1.

**Fig. 2:**
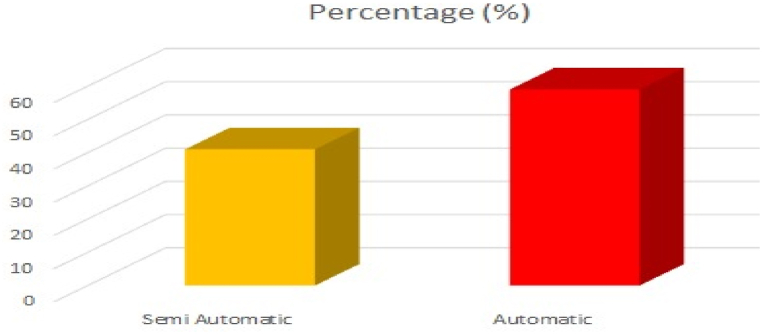
Fruits and vegetables processing market share, by mode of operations, 2021 [%].

**Fig. 3:**
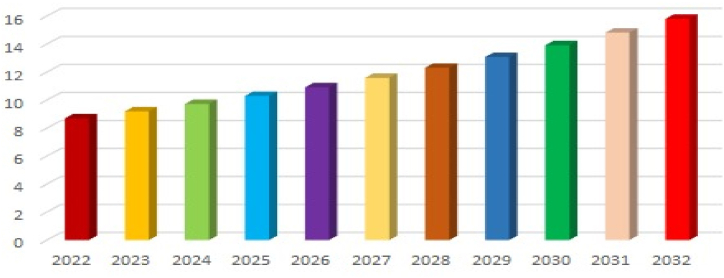
Fruits and vegetables processing market size 2022 to 2032 (USD billion $).

Accurate and efficient classification of fruits and vegetables is vital for various sectors, such as agriculture, food processing, and retail. Actually, the identification or classification of fruits/vegetables have been a laborious process, however, recently the automation has contributed to growing the productivity. Automation has additionally contributed to reduce the operational costs in this industry, particularly in response to the high labor costs in developed nations. The graphical representation of fruits and vegetables processing market share, by mode of operations as of 2021 provided by the precedence research [8] is shown in Fig. 2. In 2022, the estimated fruit and vegetable market volume at US $ was around 8 billion and by 2032, it is predicted to breach the limit of 15 billion US $. By observing the automated operations for classification and the processing market size of fruits and vegetables an accurate, effective, and efficient model for classifying fruits and vegetables is an urgency of time. As per the survey conducted by precedence research [8], the expected rise in fruits and vegetables processing market size from 2022 to 2032 is graphically presented in Fig. 3.

The study intended to suggest an accurate model for classification of fruits and vegetables through visual features. Significant amount of work [9] has been done for categorizing fruits and vegetables. The previous works uses complex CNN [10] architectures like MobileNet, InceptionV3, VGG19 and ResNet-50 [11]. It is observed that even with these complex architectures, researchers not succeeded to yield quality results and the predictions are non-interpretable even with high computational costs. The probable challenges of the study comprise complex CNN-based architectures, accuracy, efficiency, adaptability and robustness.

The study intends to suggest a simplified CNN-based architecture, with attention mechanism, with small number of convolutional layers, more efficient and effective customized pooling technique with improved feature extraction mechanism and utilizes the efficient and undiscovered optimizer for classification of fruits and vegetables. For interpreting the predictions of the proposed model, a Local Interpretable Model-Agnostic Explanation (LIME) based XAI is incorporated in the study for highlighting the prominent features of the image on the basis of which prediction has been made. We pursue to develop an automated model capable of accurately distinguish among different types of fruits and vegetables. This paper emphases to influence the abilities of CNN to enhance the accuracy and efficiency of classification of fruits and vegetables among different classes through visual features.

The main objectives of the proposed study are as follows:

• To accurately classify fruits and vegetables among variety of classes by exploiting simplified CNN architecture with attention mechanism for enhanced information focus.
• To boost the classification accuracy by integrating an efficient and reliable customized pooling technique.
• To enhance the classification speed by discovering a fast optimization algorithm.
• To incorporates an Explainable Artificial Intelligence (XAI) for interpreting the predictions of the proposed model.
• To increase the robustness and adaptability of the proposed model as compared to counterparts by manipulating the adaptive nature of optimizers.

### 1.1 Literature review

With the continuous progress in the technology, improved datasets are publicly accessible such as fruit 360 [12]. Progress in the classification of fruits and vegetables [13] through visual features is improved by using effective feature extraction techniques to configure the most prominent and desired features from an input image which enhances the performance of the model. For instance, Castro et al. [14] proposed an ensemble model by merging four popular Machine learning algorithms such as SVM, ANN, KNN and decision trees. The aim was to sort the cape gooseberry fruit among various levels by investigating the ripeness level of the fruit. During the preprocessing steps, the ensemble model was trained with RGB, L∗a∗b and HSV color spaces. It is observed, with L∗a∗b color spaces the suggested model achieves the best results. Armi and Fekri-Ershad [15] suggested improved fruit retrieval approach by exploiting texture-based feature extraction mechanism. The accuracy achieved by the recommended technique was only 90 % and the due the complexity of the architecture of the proposed model it was difficult to implement. Vijayalakshmi et al. [16] introduced a model for classification of variety of bananas. The model comprises of 5-Layer CNN architecture. The model achieves 96.98 % accuracy for the desired task. Piedad et al. [17] proposed a study on classification of bananas among different classes. The multiple algorithms were incorporated in the study like SVM and random forest. The classification task was performed on the RGB color spaces of the banana images and the optimal results were produced in the study through random forest algorithm.

Ni et al. [18], proposed an extended AlexNet model for classification of strawberries. To enhance the performance, they incorporated L2 regularization approach in CNN architecture. During preprocessing, the data augmentation techniques are applied on the images to make the model more diverse with regards to input images for predictions. It is observed that data augmentation makes a huge positive impact on the performance of a model. They obtained 90.70 % accuracy before data augmentation and an accuracy of 95.75 % is achieved by exploiting augmentation techniques for enhanced AlexNet technique they suggested. Zeng [19] suggested a study for classifying vegetables and fruits among 26 variety of classes by incorporating VGG architecture. The model achieved 95.6 % accuracy on the given task but due to the complexity of the architecture used while training and evaluating the model the computational cost of the model was high. Raisuli et al. [20] proposed a CNN model comprises of 6-layer for the categorization of three different types of dates. The benchmark models for the study were SVM and KNN. Ponce et al. [21], exploited Inception-ResNetV2 architecture for categorizing the variety of olive through visual features. The accuracy achieved by the suggested model was 95.91 % but the model was computationally expensive due to its complex structure. Asriny, Rani, and Hidayatullah [22] recommended a study to categorize oranges into 5 different classes based on oranges visual conditions. The model used in the study comprises of 4-layer CNN architecture. Similarly, Hanh and Bao [23] explored transfer learning and incorporated Yolov4 network for the classification of lemons. Nasiri et al. [24] proposed the VGG-16 architecture for the classification of dates in multiple classes. The suggested model achieves an accuracy of 96 % but the proposed model training speed was slow due to the complex architectures used while training and evaluating the model for desired purpose.

Ni et al. [25], investigated the application of transfer learning to analyze the evolving state of fruit freshness, establishing correlations between storage dates and freshness. They integrated the GoogleNet architecture to automatically excerpt features from banana images, enabling the model to acquire from input data and make accurate predictions for unseen banana images. The model reached a remarkable accuracy of 98.02 % in detection of banana freshness. Mia et al. [26] focused on the classification of six uncommon local fruits in Bangladesh. Their methodology involved resizing captured images, implementing contrast enhancement, converting the RGB color space to L∗a∗b color space, and employing k-means clustering for image segmentation. This approach yielded an impressive accuracy of 94.79 %.Sapan, N et al. [27] utilized a pre-trained CNN to categorize mangoes into different groups, employing Inception, MobileNet and DenseNet as deep learning architectures for training and comparison. The DenseNet model achieved the highest accuracy of 91.42 %. Zhang et al. [28] suggested a 13-layer CNN architecture for classification of fruits. The study incorporates data augmentation techniques during preprocessing steps which involves noise injection, rotation of images and gamma correction. The accuracy achieved by the suggested model was around 95 %.

Nagnath et al. [29], recommended CNN-based AlexNet architecture for classifying fruit and vegetables through visual features using fruit 360 dataset. The accuracy achieved by the suggested model was 81.75 %. Fu yusheng et al. [30] developed a multi-architecture model named as GoogleNet, mainly using VGGNet16 and DenseNet121 models for fruit and vegetables classification using fruit 360 dataset. The accuracies achieved by the recommended models are 95.69 % and 97.08 % for VGGNet16 and DenseNet121 respectively. Sukhuta et al. [31], exploited ResNet model for fruit and vegetable classification task on fruit 360 dataset. The accuracy achieved by the suggested model was 95.83 %.

### 1.2 Our contributions

In this study, a new simplified CNN-based variant is proposed titled as enhanced attention-convolutional neural network (EA-CNN) model for classification of fruits and vegetables through visual features using fruit 360 benchmark dataset. The proposed EA-CNN has shown significant performance as equated to the benchmark models by using simple architecture with attention mechanism, more effective pooling strategy, efficient and unexplored optimizers. The performance of the proposed novel EA-CNN in comparison with standard CNN-based variants is extremely enhanced for accurate fruits and vegetables classification through visual features. The significant contributions of the study are as follows:

• An enhanced attention-CNN model with simplified architecture providing accurate and effective outcomes for vegetables and fruits classification.
• The proposed CNN is novel in terms of simplified architecture with attention mechanism, customized pooling methods and undiscovered optimizer for solving fruits and vegetables classification task through visual features.
• The suggested model is computationally economical, achieving optimal accuracies with fewer iterations.
• The suggested research utilizes Explainable Artificial Intelligence (XAI) to explain and interpret the predictions made by the proposed model.
• The suggested model surpasses state-of-the-art (SOTA) models with reference to improved predictive accuracy for fruits and vegetables classification through visual features using standard dataset.

### 1.3 Paper organization

The workflow of the study is as follow: CNN-based Classifiers which includes popular variants used for fruits and vegetables classification task and Proposed EA-CNN architecture with attention mechanism, mixed pooling, Nadam optimizer and Explainable AI (XAI) will be discussed in Section 2. Afterwards, in Section 3 the results and discussion of the study, which comprises of dataset description, simulation environment, evaluation metrics, graphical breakdown of proposed model and comparison with benchmark models will be discussed. The predicted capabilities of the suggested model and the conclusion of the study will be discussed in 4, 5.

## 2 CNN based classifiers

### 2.1 Popular variants used for fruit and vegetable classification

#### 2.1.1 AlexNet

**Fig. 4:**
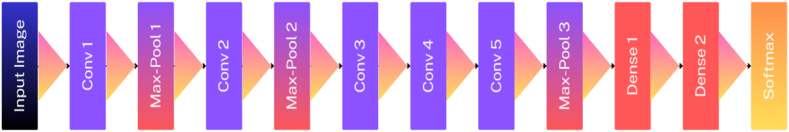
Basic architecture diagram of ALEXNET model.

ALEXNET [32] is a well-known CNN architecture used for Image classification tasks. The architecture consists of total 8 layer having 5 convolutional and 3 dense layers. ALEXNET yield multiple innovations like overlapping pooling, data augmentation and dropout regularization. The training time of ALEXNET is less because of its parallel computing capabilities. AlexNet architecture [29] has been used for fruit and vegetables classification task on fruit 360 benchmark dataset, but not the best results were produced by the recommended model. The basic architecture diagram of model is presented in Fig. 4.

#### 2.1.2 VGG16

**Fig. 5:**
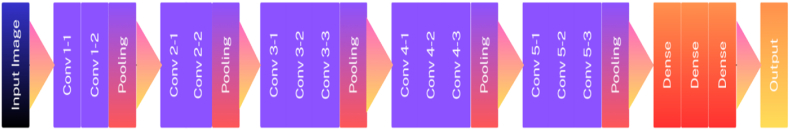
Basic structure diagram of VGG16 model.

Visual Geometry Group 16 (VGG16) is a widespread CNN architecture used for Image Classification problems. VGG 16 comprises of 16 layers having 13 convolutional layer and 3 dense layers. In all convolutional layers 3 × 3 convolutional filter been used with 2 × 2 max pooling layers. Normally Researchers [33] used pretrained models of VGG16 because of its ability of feature extraction and dimension reduction. VGG16 has also been used [30] for fruit and vegetable classification in previous related work but not the best results were produced using VGG16 architecture and computational cost of those models was also quite high. The basic structure of VGG16 is represented in Fig. 5.

#### 2.1.3 DenseNet-121

**Fig. 6:**
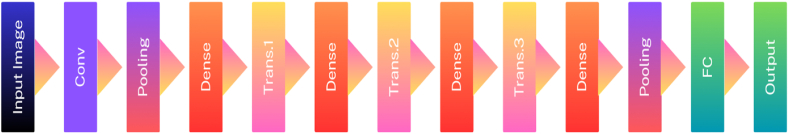
Basic structure diagram of DENSENET-121 model.

DenseNet-121 is a CNN architecture [34] which belongs to the family of DenseNets, which stands for Densely Connected Convolutional Networks. DenseNet presents a unique connectivity pattern where each layer takes direct involvement from all preceding layers. This dense connectivity results in feature reuse, inspiring the network to influence information from different depths and promoting efficient parameter usage. Fruit and vegetable classification on Fruit-360 dataset using DenseNet-121 architecture [30] has also been performed but not the best results were producing by exploiting this architecture. The basic structure diagram of DenseNet-121 is shown in Fig. 6.

### 2.2 Mathematical modeling of proposed model

The mathematical foundations behind the proposed neural architecture are given in (1), (2), (3), (4), (5), (6), (7), (8). The proposed enhanced attention-based convolutional neural network (EA-CNN) is specifically designed for fruit and vegetable classification task, where the dimension of the input image is $G\in R^{a\times b\times c}$ with height (a), width (b) and number of channels (c). Initially, the convolutional layer with 3×3 kernal size followed by rectified linear unit (ReLU) activation operation is performed, which can be mathematically expressed as:

(1) $$X_{1}=ReLU\left(E_{1}*G+w_{1}\right)$$

where $E_{1}\in R^{3\times 3\times c\times p}$ refers to the kernel weights, $w_{1}\in R^{p}$ are the biases, c shows the number of input channels (RGB in our case), p are the filters in each layer and the convolution operation is denoted by symbol ∗.

**Fig. 7:**
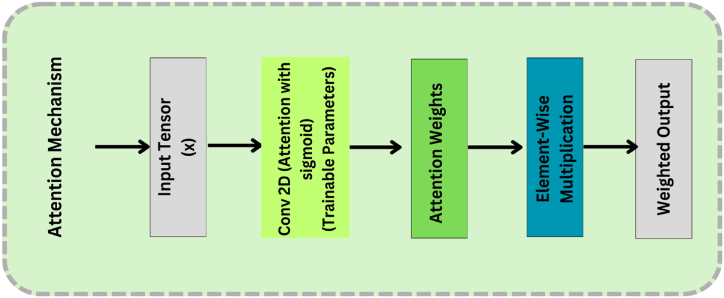
Graphical workflow of the suggested attention mechanism.

After the convolutional operation, the attention mechanism is implemented and applied to the feature maps computed through convolutional filters. The graphical workflow of the suggested attention mechanism is presented in Fig. 7. The exploited attention mechanism can be modelled as:

(2) $$M_{1}=\sigma \left(E_{i}*X_{i}+w_{i}\right)$$

where $E_{i}$ and $w_{i}$ are the corresponding weights and biases of the attention mechanism, whereas σ denotes the sigmoid function which normalizes the pixel values between 0 and 1. The attention-based prominent feature map can be expressed as:

(3) $$X_{1}^{att}=X_{1}⊙M_{1}$$

where ⊙ refers to the element wise multiplication of attention-based feature map with original input sample. Afterwards, an explored customized mixed pooling operation is applied on the output of attention mechanism which can be expressed as:

(4) $$T_{1}=Concatenate\left(MaxPooling\left(X_{1}^{att}\right),AvgPooling\left(X_{1}^{att}\right)\right)$$

The above sequence block of convolution, attention and mixed pooling is followed three times with specified filters, weights, biases, $X_{2}^{att}$, $X_{3}^{att}$*,* $T_{2}$ *,* $T_{3}$ *etc.* Beyond the final sequence block, the output is converted into single vector through flatten layer.

(5) $$k=Flatten\left(T_{3}\right)$$

Afterwards, the above vector (k) is passed to the dense architecture of the proposed EA-CNN model.

(6) $$s_{1}=SoftPlus\left(E_{k}\;.\;k+w_{k}\right)$$

Where $E_{k}$ and $w_{k}$ are the corresponding weights and biases of the dense layers. Furthermore, the dropout operation is incorporated as a regularization term.

(7) $$s_{1}^{drop}=Dropout\left(s_{1},0.5\right)$$

At last, the SoftMax function is utilized to predict the probabilities of each class.

(8) $$o^{ˆ}=SoftMax\left(E_{o}\;.\;s_{1}^{drop}+w_{o}\right)$$

where $E_{o}$ and $w_{o}$ are the final weights and biases of the output layer.

### 2.3 Proposed EA-CNN architecture

**Fig. 8:**
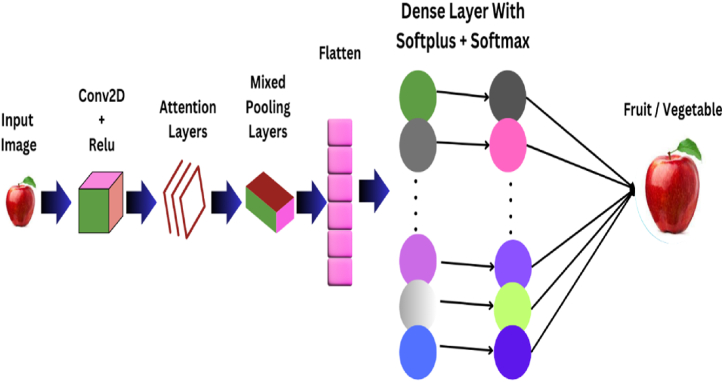
General architecture diagram of proposed EA-CNN model.

The complex task of predicting the fruit and vegetable class accurately among variety of classes through visual features is accomplished by manipulating the capability of convolutional neural network through prominent feature extraction and dimensionality reduction. The general architecture diagram of proposed Enhanced Attention-Convolutional Neural Network (EA-CNN) is represented in Fig. 8. The proposed EA-CNN model is deployed on Fruit-360 benchmark dataset, which is the biggest dataset available for desired task containing 90 k images of various fruits and vegetables. The proposed EA-CNN architecture compromises of three convolutional layers. The first layer and second layer comprise of 32∗3∗3 filter size, third layer uses 64∗3∗3 filter size along with regular CNN computations. The model uses Relu as activation function. Addition of Attention Mechanism in the proposed EA-CNN integrates Attention layer between Convolutional and pooling Layer of the architecture. The output from Convolutional layer and Relu is connected to the Attention layer and the output of Attention layer fed to Mixed Pooling layer (which is a concatenate of Max and Average pooling technique and undiscover technique for this problem) with pool size of 2∗2. Further 2 dense layers are used in the EA-CNN architecture which learn the prominent visual features of an input images of fruits and vegetables. The output of convolutional, attention and pooling layer is flattened and then fed to dense layers. The proposed model incorporates Softplus activation function in first dense layer and SoftMax activation function in the last output layer. The overall Enhanced Attention-Convolutional Neural Network (EA-CNN) architecture for Fruit and Vegetable classification on Fruit-360 standard dataset is shown in Table 1.

**Table 1:** Enhanced attention-CNN (EA-CNN) architectural parameters.

	Layer (type)	Output Shape	Parameters
Layer 1	Conv2D	None,98,98,32	896
	Activation	None,98,98,32	0
	Attention Layer	None,98,98,32	33
	Mixed Pooling2D	None,49,49,64	0
Layer 2	Conv2D_1	None,47,47,32	18464
	Activation_1	None,47,47,32	0
	Attention Layer_1	None,47,47,32	33
	Mixed Pooling2D_1	None,23,23,64	0
Layer 3	Conv2D_2	None,21,21,64	36928
	Activation_2	None,21,21,64	0
	Attention Layer_2	None,21,21,64	65
	Mixed Pooling2D_2	None,10,10,128	0
Layer 4	Flatten	None, 12800	0
	Dense	None,1024	13108224
	Activation_3	None,1024	0
	Dropout	None,1024	0
	Dense_1	None,141	134275
	Activation_4	None,141	0

Total Parameters: 13298918 (50.73 MB).

Trainable Parameters: 13298918 (50.73 MB).

Non-Trainable parameters: 0 (0.0 Byte).

#### 2.3.1 Customize mixed pooling

Mixed Pooling combines multiple operations of Average and Max pooling techniques. This approach offers several assistance in scope of deep learning:

• **Diverse Information Extraction:** Max pooling is effective at extracting most prominent information while Average pooling deliver smooth representation of entire region of input image.
• **Reduce overfitting:** Mixture of different pooling operations also contribute to regularization technique within neural network, which prevent the model to be overfitted.

#### 2.3.2 Attention feature extraction mechanism

Attention is a mechanism in representative learning that permits a model to emphasis on specific portions of input image while making predictions or decisions. Some of the benefits of exploiting attention mechanism in deep learning models is discussed below:

• **Enhanced information focus:** The attention layer permits the model to concentrate on significant parts of the input image, allowing it to evaluate inputs in a different way. This enhances the model's capability to extract most relevant information.
• **Increased robustness and adaptability:** Incorporating attention mechanisms often improves the model's robustness by reducing its sensitivity to unrelated input information. Moreover, attention weights offer a degree of adaptability, making it easier to recognize which portions of the input, the model deems most important for a given prediction.

#### 2.3.3 Nadam optimizer

Nesterov Accelerated Adaptive Moment Estimation (Nadam) concatenate advantages of both Nesterov Accelerated Gradient (NAG) and Adaptive Moment Estimation (Adam) methods. The benefits of using Nadam optimizer while training the model are as fellow:

• **Faster Convergence:** Nadam helps to converge faster towards optimal solution.
• **Adaptive Learning Rate:** Nadam utilizes adaptive learning rates for hyper parameters while training of the model.

#### 2.3.4 Explainable AI

**Fig. 9:**
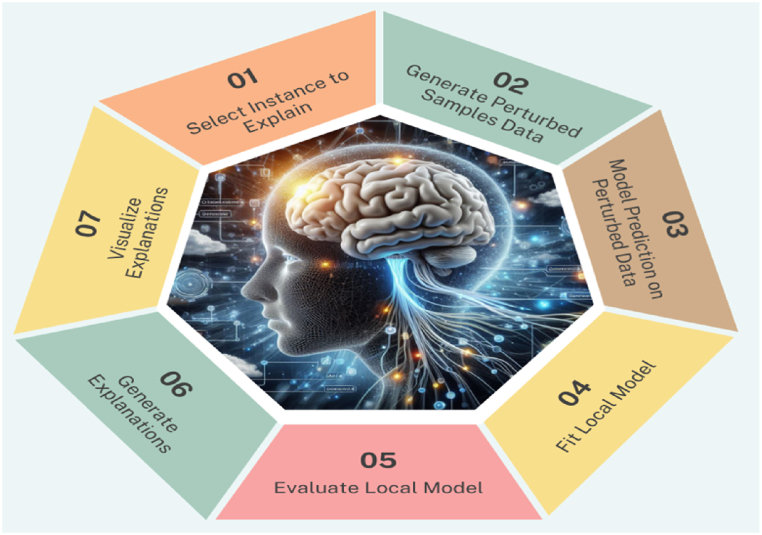
Working steps of local interpretable model-agnostic explanations (lime).

Local Interpretable Model-Agnostic Explanation (LIME) technique of Explainable AI (XAI) is exploited in the study. LIME is very helpful tool in the field of representative learning models interpretability. It is basically introduced to address the black-box nature of complex models. LIME offers a solution for producing human interpretable explanations for individual predictions. The prime aim of LIME is to offer an insight into the decision-making progression of deep learning models on a local level, providing users with a clearer understanding of why a specific prediction was made. LIME follows several steps for generating the explained predictions on any specific image by highlighting the most prominent features and their contributions behind the prediction. The graphical abstract of working process of LIME is represented in Fig. 9.

The LIME technique comprises of several key phases to make deep learning models more explainable. Initially, LIME chooses a specific instance for which an explanation is needed. This instance is perturbed to generate a dataset of similar, but slightly modified, instances. Afterwards, the complex model's predictions are attained for this perturbed dataset. In the third step, a simpler, interpretable model, such as a linear model like decision trees, is trained on the perturbed instances and their corresponding predictions. This interpretable model assists as a replacement for the complex model in the locality of the chosen instance. Lastly, the coefficients of the interpretable model are used to explain the contribution of different features, providing a human-understandable explanation for the original prediction. The LIME process basically bridges the gap between complex models and interpretability, offering valuable insights into the decision logic of deep learning algorithms.

## 3 Results and discussion

### 3.1 Dataset description

In order to validate the robustness of the proposed EA-CNN model, the experiments are conducted on two benchmark datasets. The detailed description of the databases utilized in this study are as follows:

• **Fruit-360 Dataset:**

**Fig. 10:**
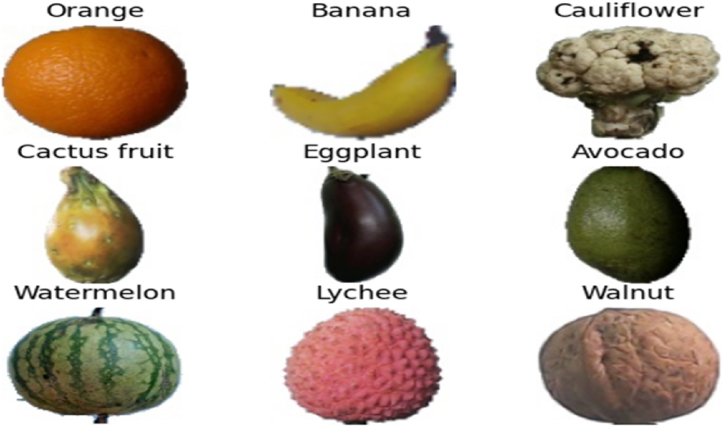
Few images of Fruit-360 benchmark dataset.

Fruit-360 [35] is one of the best publicly available database for fruit and vegetable classification task. It contains more than 90 k images across 141 different variety of fruit and vegetable classes. The wide range of variety and the quality of images in terms of resolution or uniform background in fruit-360 dataset makes it a suitable resource for designing adaptable and robust classification model. The size of images in fruit-360 dataset is 100 × 100 pixels, which maintains a good balance between quality and computational efficiency. Furthermore, as per the standard splitting ratio for deep learning models, this database is pre-split into 80:20 ration for training and testing purposes. Few of the images from different classes of fruit-360 dataset is presented in Fig. 10.

• **Fruit Recognition Dataset:**

**Fig. 11:**
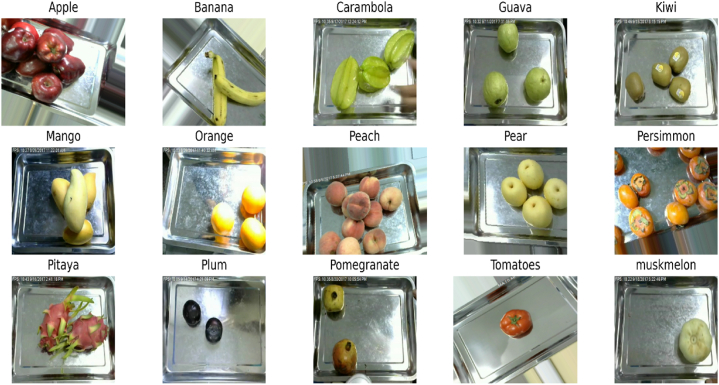
Sample images of fruit recognition dataset.

Fruit Recognition [36] dataset is specifically designed to tackle the real-life challenges faced in supermarkets. This dataset includes over 44 k images of 15 distinct fruits followed by their sub-categories. Different variations such as illumination, sunshine poses, shadow variations etc, were performed while developing this dataset to consider all the possible challenges exist in superstores. The exploitation of such diverse dataset can make the classification models reliable, robust and adaptable for real-world applications. Few sample images of fruit recognition dataset are given in Fig. 11.

### 3.2 Simulation environment

In this study, the fruits and vegetables classification task are performed on Lenovo ThinkPad laptop with 6th generation Intel core i5 processor having 8 GB RAM and 512 SSD. The Google Colab resources specifically GPUs has been used while training the model. Keras is used as deep learning framework for simulation work. These hardware and software specifications lead to the efficient model training and evaluation.

### 3.3 Evaluation metrics

The efficiency of the EA-CNN model is assessed by visualizing its accuracy in comparison to benchmark models across standard dataset. Additional metrics employed in the analysis are Mean Absolute Error (MAE), Root Mean Square Error (RMSE), and Categorical Cross Entropy Loss (CCEL) as shown in (10), (11), (9).

Mean Absolute Error (MAE) is a metric that measures the average absolute difference between predicted and actual values, providing a straightforward indication of the model's overall prediction accuracy. Mathematically, it can be expressed as fellow:

(9) $$MAE=\frac{\sum_{i=1}^{n}\left|y_{i}-x_{i}\right|}{n}\text{,}$$

where $y_{i}$ = predicted label, and $x_{i}$ = actual label

Root Mean Square Error (RMSE) is a metric that calculates the square root of the average squared differences between predicted and actual values, offering a measure of the model's ability to predict with consideration to both large and small errors. It is calculated as:

(10) $$RMSE=\frac{1}{N}\sum_{i=1}^{n}\left(\hat{X}i-Xi\right)2$$

where $\hat{X}i$ = predicted label, and Xi = actual label

Categorical Cross Entropy is a loss function commonly used in classification tasks, quantifying the dissimilarity between predicted probability distributions and actual class distributions, guiding the model towards more accurate categorization. It can be mathematically expressed as:

(11) $$L=-\frac{1}{N}\sum_{i=1}^{N}=\sum_{j=1}^{C}=y_{ij}\;\log\left(p_{ij}\right)$$

### 3.4 Results and discussion

In this section, the outcomes concluded from the experiments of proposed EA-CNN model on both benchmark datasets will be critically analyzed.

#### 3.4.1 Study-I (with Fruit-360 dataset)

**Fig. 12:**
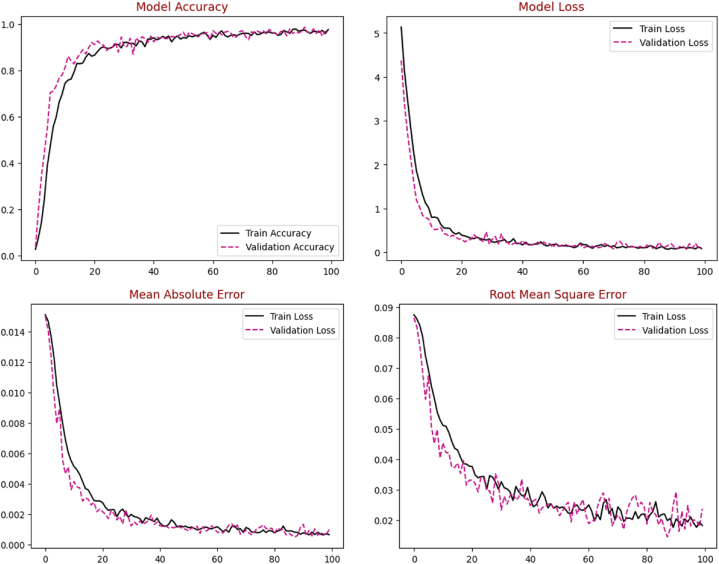
Learning behavior of proposed EA-CNN model on Fruit-360 dataset.

**Fig. 13:**
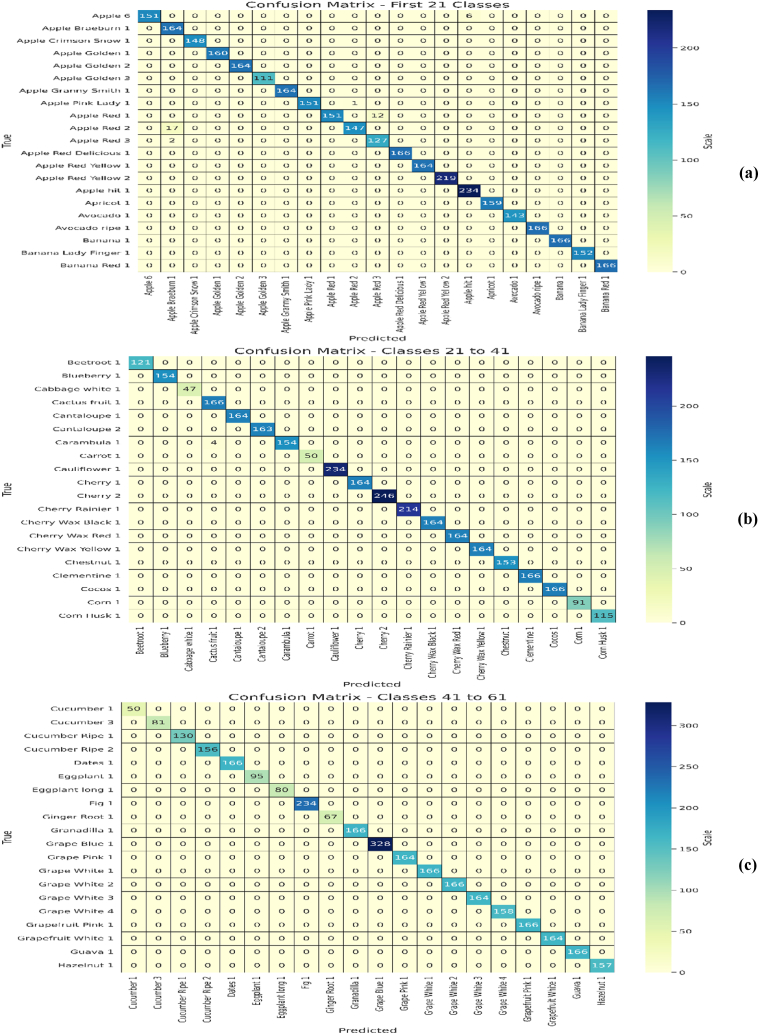

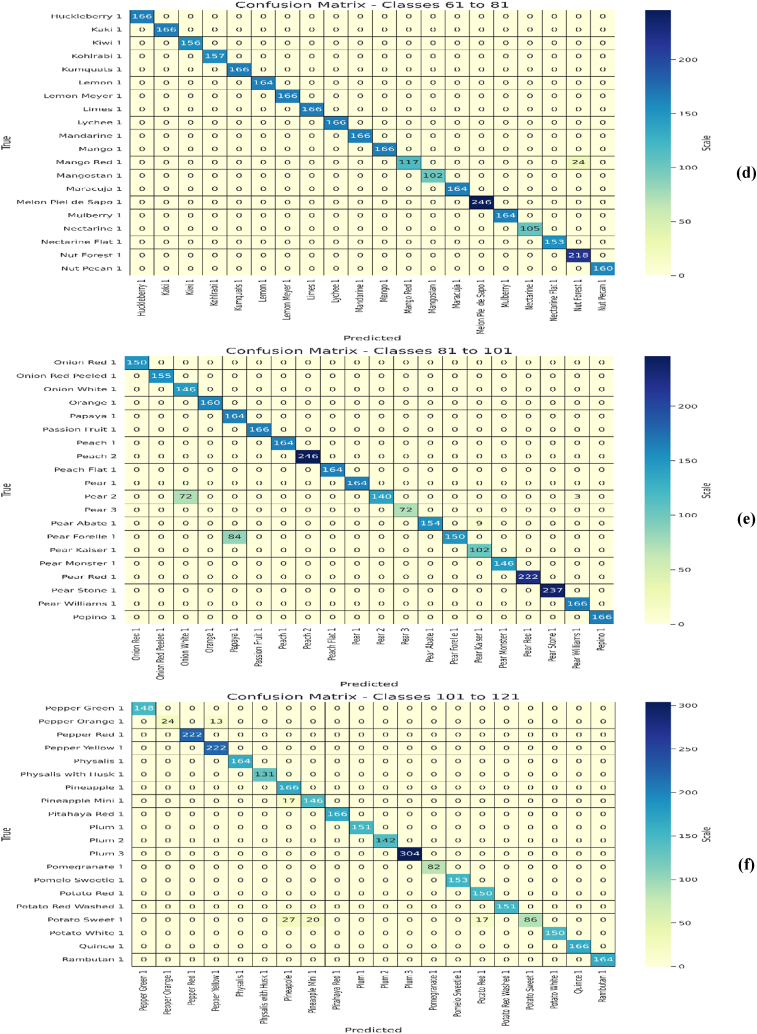

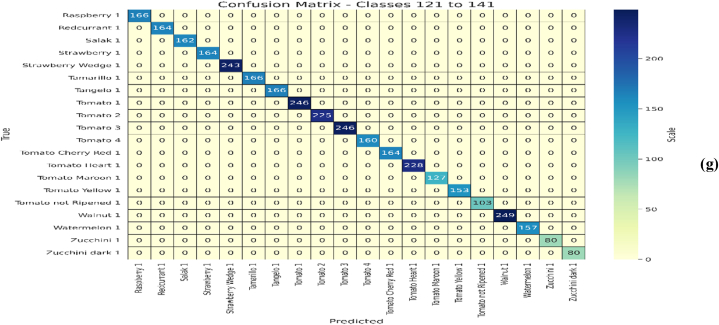
Performance analysis of EA-CNN model on Fruit-360 test dataset through confusion matrixes; (a) for first 21 classes, (b) for classes 21 to 41, (c) for classes 41 to 61, (d) for classes 61 to 81, (e) for classes 81 to 101, (f) for classes 101 to 121, (g) for classes 121 to 141.

Initially, the proposed EA-CNN model is trained using Fruit-360 benchmark dataset with the ratio of 70 % training and 30 % testing data. Due to improved attention mechanism and mixed pooling technique better feature extraction is observed which leads to remarkable results by the proposed EA-CNN. Furthermore, the model converges quickly by applying Nadam optimizer, which strengthen the model to achieve optimal results after utilizing only 100 epochs. The proficiency of the model with regards to accuracy, Loss, MAE and RMSE is represented in Fig. 12. The dataset contains 141 classes and visualizing all these classes in single confusion matrix is not possible, for that purpose we have included each confusion matrices of 20 classes separately in the study. The confusion matrixes generated for the model can be visualized in Fig. 13(a–g). The proposed EA-CNN model achieves a test accuracy of 98.1 % and outperformed the benchmark models. Furthermore, in order to provide the detailed statistical performance analysis of proposed EA-CNN model on fruit-360 dataset, an extensive classification report is given in Table .2.

**Table 2:** Statistical performance evaluation of EA-CNN model on Fruit-360 test data is provided through comprehensive classification report.

**Dataset Labels**	**Precision**	**Recall**	**F1-Score**
Apple 6	1.00	0.96	0.98
Apple Braeburn 1	0.51	1.00	0.67
Apple Crimson Snow 1	1.00	1.00	1.00
Apple Golden 1	0.75	1.00	0.86
Apple Golden 2	1.00	1.00	1.00
Apple Golden 3	1.00	0.69	0.82
Apple Granny Smith 1	1.00	1.00	1.00
Apple Pink Lady 1	0.94	0.99	0.97
Apple Red 1	1.00	0.92	0.96
Apple Red 2	0.98	0.90	0.94
Apple Red 3	0.91	0.88	0.90
Apple Red Delicious 1	1.00	1.00	1.00
Apple Red Yellow 1	1.00	1.00	1.00
Apple Red Yellow 2	1.00	1.00	1.00
Apple hit 1	0.97	1.00	0.99
Apricot 1	0.45	0.97	0.61
Avocado 1	1.00	1.00	1.00
Avocado ripe 1	1.00	1.00	1.00
Banana 1	1.00	1.00	1.00
Banana Lady Finger 1	1.00	1.00	1.00
Banana Red 1	1.00	1.00	1.00
Beetroot 1	1.00	0.81	0.89
Blueberry 1	1.00	1.00	1.00
Cabbage white 1	1.00	1.00	1.00
Cactus fruit 1	0.89	1.00	0.94
Cantaloupe 1	1.00	1.00	1.00
Cantaloupe 2	0.96	0.99	0.98
Carambula 1	1.00	0.93	0.96
Carrot 1	1.00	1.00	1.00
Cauliflower 1	0.88	1.00	0.94
Cherry 1	1.00	1.00	1.00
Cherry 2	1.00	1.00	1.00
Cherry Rainier 1	1.00	0.87	0.93
Cherry Wax Black 1	1.00	1.00	1.00
Cherry Wax Red 1	1.00	1.00	1.00
Cherry Wax Yellow 1	1.00	1.00	1.00
Chestnut 1	0.87	1.00	0.93
Clementine 1	1.00	1.00	1.00
Cocos 1	1.00	1.00	1.00
Corn 1	0.74	0.61	0.67
Corn Husk 1	1.00	0.75	0.86
Cucumber 1	1.00	1.00	1.00
Cucumber 3	1.00	1.00	1.00
Cucumber Ripe 1	1.00	1.00	1.00
Cucumber Ripe 2	1.00	1.00	1.00
Dates 1	1.00	1.00	1.00
Eggplant 1	1.00	0.61	0.76
Eggplant long 1	1.00	1.00	1.00
Fig. 1	1.00	1.00	1.00
Ginger Root 1	1.00	0.68	0.81
Granadilla 1	1.00	1.00	1.00
Grape Blue 1	1.00	1.00	1.00
Grape Pink 1	0.91	1.00	0.95
Grape White 1	1.00	1.00	1.00
Grape White 2	1.00	1.00	1.00
Grape White 3	1.00	1.00	1.00
Grape White 4	1.00	1.00	1.00
Grapefruit Pink 1	1.00	1.00	1.00
Grapefruit White 1	1.00	1.00	1.00
Guava 1	1.00	1.00	1.00
Hazelnut 1	1.00	1.00	1.00
Huckleberry 1	1.00	1.00	1.00
Kaki 1	1.00	1.00	1.00
Kiwi 1	1.00	1.00	1.00
Kohlrabi 1	1.00	1.00	1.00
Kumquats 1	1.00	1.00	1.00
Lemon 1	1.00	1.00	1.00
Lemon Meyer 1	1.00	1.00	1.00
Limes 1	1.00	1.00	1.00
Lychee 1	1.00	1.00	1.00
Mandarine 1	1.00	1.00	1.00
Mango 1	1.00	1.00	1.00
Mango Red 1	1.00	0.82	0.90
Mangostan 1	1.00	1.00	1.00
Maracuja 1	1.00	0.99	0.99
Melon Piel de Sapo 1	0.98	1.00	0.99
Mulberry 1	1.00	1.00	1.00
Nectarine 1	1.00	0.64	0.78
Nectarine Flat 1	1.00	0.96	0.98
Nut Forest 1	0.90	1.00	0.95
Nut Pecan 1	1.00	0.90	0.95
Onion Red 1	1.00	1.00	1.00
Onion Red Peeled 1	1.00	1.00	1.00
Onion White 1	0.67	1.00	0.80
Orange 1	0.97	1.00	0.98
Papaya 1	0.66	1.00	0.80
Passion Fruit 1	1.00	1.00	1.00
Peach 1	1.00	1.00	1.00
Peach 2	1.00	1.00	1.00
Peach Flat 1	1.00	1.00	1.00
Pear 1	0.77	1.00	0.87
Pear 2	1.00	0.60	0.75
Pear 3	1.00	1.00	1.00
Pear Abate 1	1.00	0.93	0.96
Pear Forelle 1	0.99	0.64	0.78
Pear Kaiser 1	0.83	1.00	0.91
Pear Monster 1	0.97	0.88	0.92
Pear Red 1	1.00	1.00	1.00
Pear Stone 1	0.84	1.00	0.92
Pear Williams 1	0.98	1.00	0.99
Pepino 1	0.83	1.00	0.91
Pepper Green 1	0.72	1.00	0.84
Pepper Orange 1	1.00	0.10	0.19
Pepper Red 1	1.00	1.00	1.00
Pepper Yellow 1	0.94	1.00	0.97
Physalis 1	1.00	1.00	1.00
Physalis with Husk 1	0.96	0.80	0.87
Pineapple 1	0.69	1.00	0.82
Pineapple Mini 1	0.88	0.90	0.89
Pitahaya Red 1	1.00	1.00	1.00
Plum 1	1.00	1.00	1.00
Plum 2	1.00	1.00	1.00
Plum 3	0.94	1.00	0.97
Pomegranate 1	1.00	0.50	0.67
Pomelo Sweetie 1	1.00	1.00	1.00
Potato Red 1	0.90	1.00	0.95
Potato Red Washed 1	1.00	1.00	1.00
Potato Sweet 1	1.00	0.57	0.73
Potato White 1	0.98	1.00	0.99
Quince 1	1.00	1.00	1.00
Rambutan 1	1.00	1.00	1.00
Raspberry 1	1.00	1.00	1.00
Redcurrant 1	1.00	1.00	1.00
Salak 1	1.00	1.00	1.00
Strawberry 1	1.00	1.00	1.00
Strawberry Wedge 1	1.00	0.99	0.99
Tamarillo 1	1.00	1.00	1.00
Tangelo 1	1.00	1.00	1.00
Tomato 1	0.97	1.00	0.99
Tomato 2	1.00	1.00	1.00
Tomato 3	1.00	1.00	1.00
Tomato 4	1.00	1.00	1.00
Tomato Cherry Red 1	1.00	1.00	1.00
Tomato Heart 1	1.00	1.00	1.00
Tomato Maroon 1	1.00	1.00	1.00
Tomato Yellow 1	1.00	1.00	1.00
Tomato not Ripened 1	1.00	0.65	0.79
Walnut 1	1.00	1.00	1.00
Watermelon 1	0.87	1.00	0.93
Zucchini 1	1.00	1.00	1.00
Zucchini dark 1	1.00	1.00	1.00

#### 3.4.2 Study-II (with fruit recognition dataset)

**Fig. 14:**
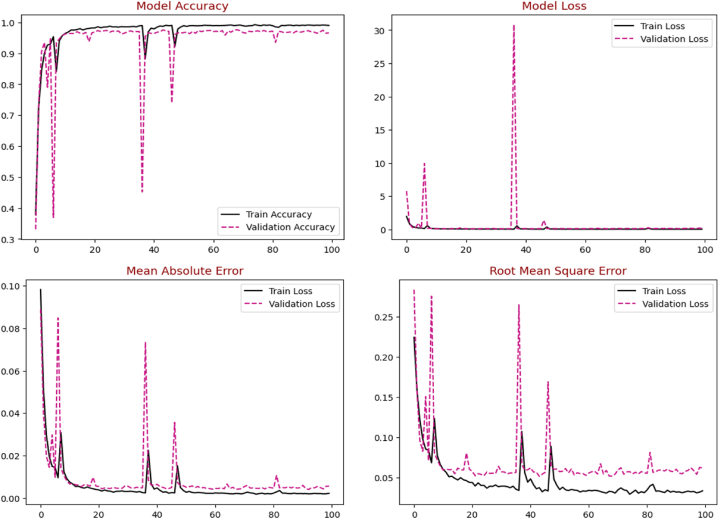
Learning behavior of proposed EA-CNN model on fruit recognition dataset.

**Fig. 15:**
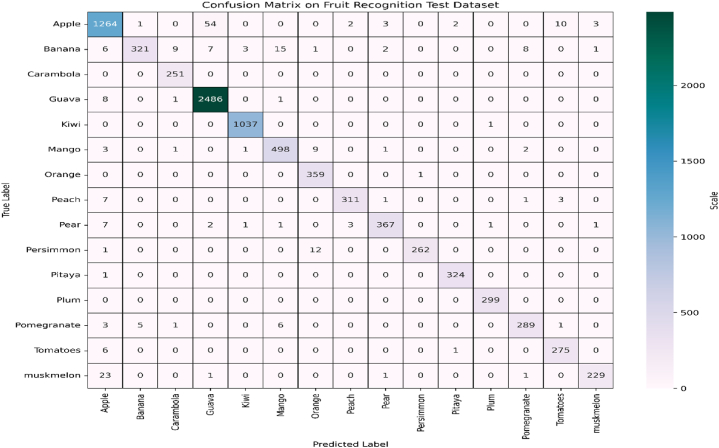
Performance analysis of EA-CNN model on fruit-recognition test dataset through confusion matrix.

**Fig. 16:**
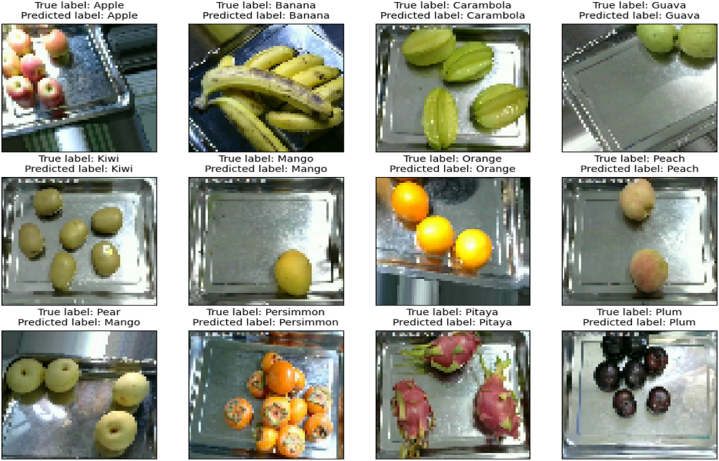
Inference capabilities of proposed EA-CNN model on fruit-recognition test dataset.

In order to verify the robustness, scalability and adaptability of the proposed EA-CNN model, another standard fruit recognition dataset is exploited in this study. The proposed EA-CNN model is executed with a division ratio of 75:25 for training and testing purposes. Due to the effective feature extraction process of the proposed model, it shows substantial performance in terms of generalized accuracy and fast convergence speed for this dataset aswell. The graphical performance analysis of the proposed EA-CNN model in terms of learning behavior is shown in Fig. 14. The confusion matrix of the model on the test dataset is given in Fig. 15. The statistical performance of the proposed model is analyzed through effective evaluation metrics such as precision, recall, f1-score and accuracy. The resulted classification report of the proposed EA-CNN model on test data is given in Table .3. The proposed model reaches a generalized accuracy of 96 % on test fruit recognition data and outperformed the benchmark models. The inference capabilities of the model on the test dataset are presented in Fig. 16. The above discussion proves the effectiveness, robustness, scalability and diversity of the EA-CNN model which shows substantial classification performance on the both the benchmark datasets.

**Table 3:** Statistical performance evaluation of EA-CNN model on fruit-recognition test data is provided through comprehensive classification report.

**Dataset Labels**	**Precision**	**Recall**	**F1-Score**
Apple	0.95	0.94	0.95
Banana	0.98	0.86	0.92
Carambola	0.95	1.00	0.98
Guava	0.97	1.00	0.99
Kiwi	1.00	1.00	1.00
Mango	0.96	0.97	0.96
Orange	0.94	1.00	0.97
Peach	0.98	0.96	0.97
Pear	0.98	0.96	0.97
Persimmon	1.00	0.95	0.97
Pitaya	0.99	1.00	0.99
Plum	0.99	1.00	1.00
Pomegranate	0.96	0.95	0.95
Tomatoes	0.95	0.98	0.96
muskmelon	0.98	0.90	0.94

#### 3.4.3 Comparison with benchmark classification models

To prove the effectiveness of the proposed EA-CNN model over benchmark models, the comparative analysis of the proposed model with state-of-the-art (SOTA) models using two standard datasets like fruit-360 and fruit recognition is performed in this section. The description of the benchmark models is given below:

• **Attention-based MobileNetV2:** The [37] convolutional block attention module (CBAM) is incorporated within lightweight pre-trained MobileNetV2 architecture.
• **CAE-AND:** In Ref. [38], a hybrid architectural framework of attention-based convolutional network and convolutional encoder is designed for fruit and vegetable classification task.
• **MobileNet-SE and Xception-SE:** The squeeze and excitation (SE)-based [39] attention mechanism is incorporated as additional layer in the MobileNet and Xception architectures.
• **Transfer Learning (TL):** In Ref. [40], the TL-based pre-trained ResNEt-50 and MobileNEtV3Small is exploited for fruit recognition task.

**Fig. 17:**
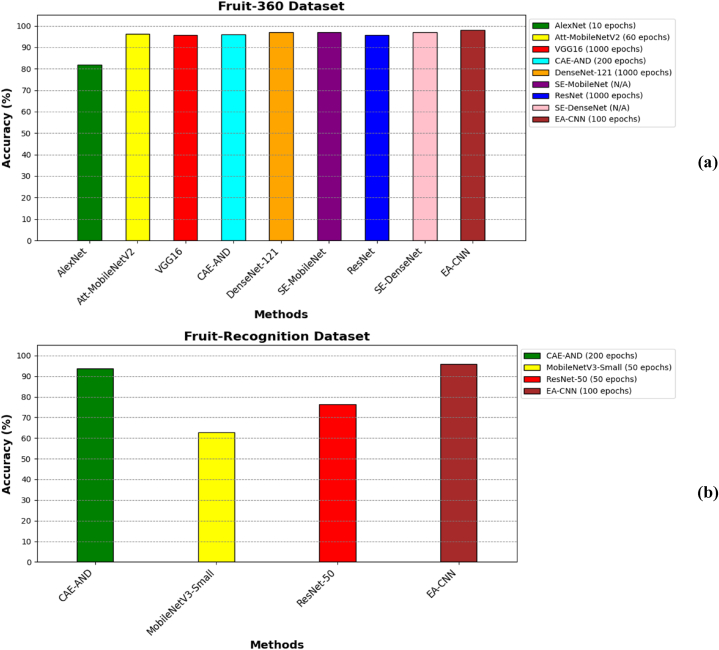
Graphical performance assessment of proposed EA-CNN model with benchmark models on; (a) Fruit-360 dataset, (b) fruit recognition dataset.

Evaluation of the proposed EA-CNN in terms of accuracy against the benchmark models is enlisted in Table .4. It is depicted that the suggested model has outclasses its counterparts in terms of accuracy, computational cost and scalability. The graphical assessment of the model with benchmark models can be visualized in Fig. 17(a and b).

**Table 4:** Comparitive analysis of EA-CNN model with existing attention-based and other stata-of-the-art models on chosen benchmark datasets.

**METHODS**	**DATASET**	**CLASSES**	**EPOCHS**	**ACCURACY (%)**
AlexNet [29]	Fruit-360	18	10	81.75
Att-MobileNetV2 [37]	Fruit-360	53	60	96.2
VGG16 [30]	Fruit-360	–	1000	95.69
CAE-AND [38]	Fruit-360	26	200	95.86
DenseNet-121 [30]	Fruit-360	–	1000	97.08
SE-MobileNet [39]	Fruit-360	36	–	97.0
ResNet [31]	Fruit-360	06	1000	95.8
SE-DenseNet [39]	Fruit-360	36	–	97.0
**EA-CNN (proposed)**	**Fruit-360**	**141**	**100**	**98.1**
ResNet-50 [40]	Fruit Recognition	15	50	76.47
CAE-AND [38]	Fruit Recognition	15	200	93.78
MobileNetV3-Small [40]	Fruit Recognition	15	50	62.73
**EA-CNN (proposed)**	**Fruit-Recognition**	**15**	**100**	**96**

## 4 Predictive capability on Fruit-360 benchmark dataset

**Fig. 18:**
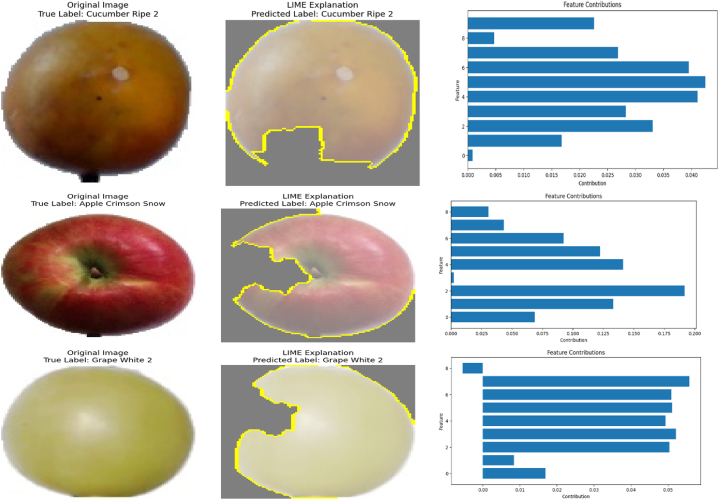

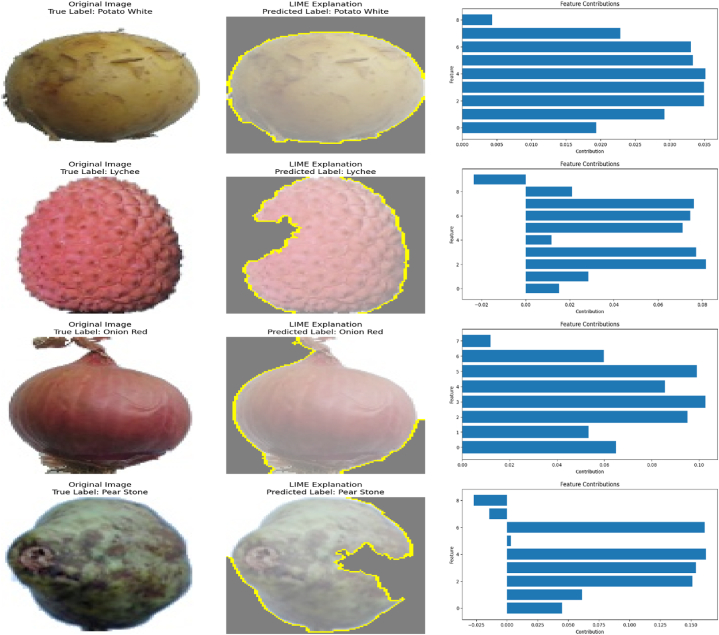
Explainable predictive capabilities of proposed EA-CNN model.

The proposed EA-CNN model demonstrates significant predictive capabilities in efficiently and accurately classifying fruits and vegetables. We have extensively trained the proposed EA-CNN model on the dataset and validated the reliable predictive performance of the proposed model through rigorous testing. The proposed model effectively distinguishes the different product types. In order to make the proposed model interpretable, we have incorporated the Local Interpretable Model-Agnostic Explanation (LIME)-based XAI approach for highlighting the prominent features in the analyzed images. Some of the predictions of the proposed EA-CNN model on unseen images from the benchmark dataset are provided in Fig. 18 with the visualization of explainable predictions. From Fig. 18, it is noted that three different aspects of demonstration are provided for each specified prediction. Initially, the original unseen image with ground truth is provided as a reference point. Secondly, the LIME explanation along predicted label is shown to offer the influential insights about the predictive behavior of the proposed EA-CNN model. The aim of LIME is to identify the most prominent regions or features in the input image. The yellow highlighted regions in Fig. 18, shows the influence of input features behind the decision-making process of the EA-CNN model. At last, the contributions of the most prominent features (segments/super pixels) are graphically presented through bar charts for providing ease in the understandability of the model's prediction scheme. The visualizations in Fig. 18, are presented to provide the interpretable, transparent and human understandable behavior of the proposed EA-CNN model. It is seen that these visualizations give a clear understanding of the model's interpretability and its ability to explain predictions in the context of product classification.

## 5 Conclusions

The findings obtained from the study are as follows: The suggested EA-CNN approach contributes as a remarkable addition in fruits and vegetables classification through visual features. The proposed EA-CNN demonstrates noteworthy predictive capabilities in efficiently and accurately classifying fruits and vegetables. The better accuracy achieved by the proposed EA-CNN as compared to its counterparts on benchmark datasets, verify the effectiveness, adaptability, robustness and scalability of EA-CNN over benchmark models. The proposed CNN model has attained a generalized test accuracies of 98.1 % and 96 % on benchmark fruit-360 and fruit recognition databases. By incorporating XAI, the proposed EA-CNN generates a human-understandable predictions on the test images, which proves the interpretability of the proposed work. The robustness of the model in fruits and vegetables classification indicates its adaptability to various circumstances, making it a valuable tool for market places and in retail business where automation has seen a rapid increase. In the future, with more effective or novel pooling technique and with better optimizers, a more efficient architecture can be implemented to enhance performance.

## CRediT authorship contribution statement

**Zeshan Aslam Khan:** Writing – original draft, Validation, Conceptualization. **Muhammad Waqar:** Writing – original draft, Methodology. **Khalid Mehmood Cheema:** Visualization, Project administration, Funding acquisition. **Ali Abu Bakar Mahmood:** Writing – original draft. **Quratul Ain:** Visualization, Validation. **Naveed Ishtiaq Chaudhary:** Writing – review & editing. **Abdullah Alshehri:** Project administration, Funding acquisition. **Sultan S. Alshamrani:** Project administration, Funding acquisition. **Muhammad Asif Zahoor Raja:** Writing – review & editing.

## Data availability statement

Data exploited in conducted research is referenced [35,36] in this research article.

## Funding

This research was funded by 10.13039/501100006261Taif University, Saudi Arabia, Project No. (TU-DSPP-2024-52).

## Declaration of competing interest

The authors declare that they have no known competing financial interests or personal relationships that could have appeared to influence the work reported in this paper.
